# Enhancing Ablation Resistance of TaB_2_-Based Ultra-High Temperature Ceramics by Mixing Fine TaC Particles and Dispersed Multi-Walled Carbon Nanotubes

**DOI:** 10.3390/ma17143394

**Published:** 2024-07-09

**Authors:** Guangxu Bo, Xiaoke Tian, Huanhuan Li, Luona Ye, Xiaoling Xu, Zhaorui Gu, Jinyong Yan, Xingjian Su, Yunjun Yan

**Affiliations:** Key Laboratory of Molecular Biophysics of the Ministry of Education, College of Life Science and Technology, Huazhong University of Science and Technology, Wuhan 430074, China; b13991359@163.com (G.B.); d202280835@hust.edu.cn (X.T.); 18739976787@163.com (H.L.); yeln1992@163.com (L.Y.); xxl101010@126.com (X.X.); hustgzr@hust.edu.cn (Z.G.); yjiny@126.com (J.Y.); suxingjian@hust.edu.cn (X.S.)

**Keywords:** TaB_2_-based ultra-high temperature ceramics, ablation resistance, physical pinning effect, sealing effect, self-healing effect

## Abstract

Ultra-high temperature ceramics (UHTCs) have been widely applied in many fields. In order to enhance the comprehensive properties of TaB_2_-based UHTCs, the first collaborative use of fine TaC particles and dispersed multi-walled carbon nanotubes (MWCNTs) was employed via spark plasma sintering (SPS) at 1700 °C. The derived UHTCs exhibited an average grain size of 1.3 μm, a relative density of 98.6%, an elastic modulus of 386.3 GPa, and a nano hardness of 21.7 GPa, leading to a greatly improved oxidation resistance with a lower linear ablation rate at −3.3 × 10^−2^ μm/s, and a markedly reinforced ablation resistance with mass ablation rate of −1.3 × 10^−3^ mg/(s·cm^2^). The enhanced ablation resistance was attributable to the physical pinning effect, sealing effect and self-healing effect. Thus, this study provides a potential strategy for preparation of UHTCs with bettered ablation resistance and physical properties.

## 1. Introduction

Ultra-high temperature ceramics (UHTCs) have been widely applied in thermo-chemically harsh environments, such as hypersonic flights, rocket propulsion, and atmospheric re-entry, because of their high hardness, high melting point, outstanding chemical stability, excellent thermal stability, and sufficient elastic modulus [1,2]. The raw materials of UHTCs generally include carbides, nitrides, and diborides of early transition metals [3,4].

As a crucial part of UHTCs, transition metal diborides have been widely concerned with physical properties, oxidation resistance, ablation resistance, acid and alkali corrosion resistance, etc. Neuman et al. [5] and Xu et al. [6], respectively, reported dense ZrB_2_ ceramics by adding sintering aids (including carbon and boron carbide nanopowder) and refined particles to improve the mechanical and oxidation-resistant properties. Mattia et al. [7] performed oxidation studies of an aluminum nitride-hafnium diboride ceramic composite, and discovered that its anti-oxidant properties were enhanced by the formation of a protective oxide scale containing hafnia (HfO_2_) and aluminum borate (Al_18_B_4_O_33_) phases. Monticelli et al. [8] synthesized an HfB_2_-2.5 wt% Si_3_N_4_ ceramic via hot pressing sintering, and further exhibited a good corrosion behavior in acid and alkaline solutions of chlorides and sulfates. Hassan et al. [9] fabricated SiC reinforced HfB_2_ + ZrB_2_ composites with or without CNTs using spark plasma sintering (SPS), and revealed that CNTs’ reinforcement increased the heterogeneity and consequently gave rise to the formation of a wide spectrum of solid solutions of varying composition.

Compared to the widely reported ZrB_2_ and HfB_2_, TaB_2_ possesses a high melting point, high hardness, good anti-oxidation, and fine chemical attack resistance. However, it has received less attention and few studies have been reported [10,11]. Due to the strong covalent bonds, high melting point, or low self-diffusion coefficients, pure TaB_2_ gains relatively low sinterability. Thus, in addition to high temperature and high pressure, sintering aids are usually required to achieve high density. But a large-amount usage of typical sintering aid, namely SiC, not only alters the properties of UHTCs, but also introduces unnecessary complex secondary phases [3].

As is known, owing to its refinement of grain size and physical pinning effect, TaC is often selected as an ideal strengthening particle to reinforce the mechanical properties of UHTCs [12]. On the other hand, dispersed multi-walled carbon nanotubes (MWCNTs) with sealing effect can help to reinforce ablation resistance and physical properties of UHTCs [9]. Therefore, in this study, in order to improve the poor sintering behavior of TaB_2_ and further enhance physical properties and ablation resistance of TaB_2_-based HUTCs, the collaborative use of fine TaC particles and dispersed MWCNTs was first employed to synthesize TaB_2_-based UHTCs using SPS. Based on the reported literature and considering production cost reduction, the large size of TaB_2_ (1.0–3.0 μm) was chosen as a raw material for synthesizing TaB_2_-based HUTCs, and 5.0 wt% of small TaC particles (about 0.3 μm), and 0.5 wt% of MWCNTs were collaboratively utilized as reagents for enhancing the comprehensive properties of the TaB_2_-based UHTCs, and 0.2 wt% of poly(acrylic acid) (PAA) was simultaneously added to improve the dispersibility of the MWCNTs. The physical properties, high-temperature oxidation resistance, and ablation resistance of the above-derived TaB_2_-based HUTCs were further investigated using a nano-indentation instrument, a field emission scanning electron microscopy (FESEM), an X-ray diffraction (XRD) equipment, a thermogravimetric analyzer (TGA), and a welding torch. Furthermore, the mechanism of the reinforced corrosion resistance of the TaB_2_-based UHTCs using mixing TaC and MWCNTs was analyzed and proposed.

## 2. Materials and Methods

### 2.1. Materials

Tantalum diboride (TaB_2_) powder was purchased from Hunan Huawei Jingcheng Material Technology Co., Ltd., Changsha, China, with a purity of 99.5% and a size distribution of 1.0–3.0 μm. Tantalum carbide (TaC) powder was bought from Qinhuangdao Yinuo High-tech Material Development Co., Ltd., Qinghuangdao, China, with a purity of 99.0% and an average particle size of 0.3 μm. Multi-walled carbon nanotubes (MWCNTs) (>97.0%, tube length = 15.0–30.0 μm, tube diameter = 3–15 nm) were commercially obtained from Shenzhen Turin Evolution Technology Co., Ltd., Shenzhen, China. Poly(acrylic acid) (PAA) (solid content 30.0%, average molecular weight = 3000) was commercially obtained from Shanghai Maclin Biochemical Technology Co., Ltd., Shanghai, China. Anhydrous ethanol was bought from China National Pharmaceutical Group Co., Ltd., Beijing, China.

### 2.2. Powder Processing

Pure TaB_2_ powder: 12 g of TaB_2_ and 200 mL of anhydrous ethanol were added into a 500 mL glass beaker, and then the beaker was placed on a magnetic stirrer with 400 rpm for 5 h at 30 °C. Then, the reagents in the beaker were pumped into a filter device. The filtrated reagents were transferred into a new 500 mL glass beaker, then 200 mL of anhydrous ethanol was added into the glass beaker, and then the beaker was placed in an ultrasonic bath for 3 h of ultrasound. The reagents in the beaker were poured into a 500 mL single-mouth flask, which was then placed in a rotary evaporator to remove anhydrous ethanol and obtain dry TaB_2_. The dried TaB_2_ was placed into an agate mortar for grinding the apparent lumps into powders. During the rotary evaporation, the collected anhydrous ethanol was used for the processing of the next powder.

95TaB_2_-5TaC powder: The mass of TaB_2_ was 11.4 g and that of TaC was 0.6 g. The powder processing method followed the same procedure as that of the pure TaB_2_ powder.

95TaB_2_-5TaC-0.5MWCNTs powder: The mass of TaB_2_ was 11.4 g, that of TaC was 0.6 g, and that of MWCNTs was 0.06 g. The powder processing method was the same as that of the pure TaB_2_ powder.

95TaB_2_-5TaC-0.5MWCNTs-0.2PAA powder: The mass of TaB_2_ was 11.4 g, that of TaC was 0.6 g, that of MWCNTs was 0.06 g, and the mass of PAA was 0.024 g. The powder processing method was also as the same as the pure TaB_2_ powder.

### 2.3. Spark Plasma Sintering (UHTCs Preparation)

A graphite punch with a diameter of 20 mm was inserted into a graphite die with a 20 mm in inner diameter, and a circular carbon paper with a diameter of 20 mm was placed on the upper surface of the graphite punch. In total, 12 g of pure TaB_2_ powder was poured into the graphite die. A mechanical force of 8 MPa was applied to the graphite die with graphite punches inserted above and below to the compact pure TaB_2_ powder.

Graphite felt with 5 mm thick was wrapped around the outer surface of the graphite die to evenly distribute temperature and reduce heat loss from radiation. The graphite die wrapped with the graphite felt was placed in a spark plasma sintering (SPS) device (LABOX-1575, Sinter Land, Nagaoka, Japan) equipped with a thermometer (emissivity 0.96). Axial pressure of 20 MPa was applied to ensure adequate electrical contact between the pure TaB_2_ powder and the graphite die. When the vacuum degree was less than 30 Pa, the pure TaB_2_ powder was heated from room temperature with a heating rate at 100 °C/min, and the axial pressure was increased with 2.5 MPa/min. When the temperature reached 800 °C, the axial pressure was 40 MPa, and the SPS device was kept off the heat for 1 min. Then, the axial pressure remained at 40 MPa and the graphite die was continued to be heated to 1700 °C with a heating rate of 100 °C/min. After being kept at 1700 °C for 5 min, the vacuum degree and axial pressure were released, and the graphite die was cooled to room temperature rapidly. The graphite felt, the graphite die, and the carbon papers were removed to obtain the ultra-high temperature ceramic (UHTC), which was named pure TaB_2_. 95TaB_2_-5TaC, 95TaB_2_-5TaC-0.5MWCNTs, and 95TaB_2_-5TaC-0.5MWCNT-0.2PAA were, respectively, obtained as the same as that of the pure TaB_2_. The four UHTCs were about 20 mm in diameter and 4 mm in thickness.

The SPS curves of the four UHTCs are shown in Figure S1. The top view and main view of the four UHTCs are present in Figure S2.

### 2.4. Characterization

The field emission scanning electron microscope (FESEM) images of the pure TaB_2_ powder, 95TaB_2_-5TaC powder, 95TaB_2_-5TaC-0.5MWCNTs powder, and 95TaB_2_-5TaC-0.5MWCNTs-0.2PAA powder were, respectively, obtained using FESEM (Nova NanoSEM 450, FEI Company, Eindhoven, The Netherlands). The powders were, respectively, sprayed with a thin layer of platinum before being examined using FESEM.

X-ray diffraction (XRD) data of the pure TaB_2_ powder, 95TaB_2_-5TaC powder, 95TaB_2_-5TaC-0.5MWCNTs powder, and 95TaB_2_-5TaC-0.5MWCNTs-0.2PAA powder were, respectively, gained using XRD equipment (x’pert3 powder, Panaco Company, Almelo, The Netherlands) using CuKα radiation (λ = 0.154 nm) at 40 mA and 40 kV in the range 2θ = 10–90° lasting 10 min.

The relative density of the UHTCs, including the pure TaB_2_, 95TaB_2_-5TaC, 95TaB_2_-5TaC-0.5MWCNTs, and 95TaB_2_-5TaC-0.5MWCNTs-0.2PAA, were tested using the Archimedes’ method by considering the theoretical values for TaB_2_ and TaC equal to 12.6 g/cm^3^ [13] and 14.6 g/cm^3^ [14], respectively.

Elastic modulus and nano-scale hardness of all UHTCs were, respectively, tested using an Agilent Nano Indenter (G200, Keysight Technologies, Santa Rosa, CA, USA) with an 8000 μN applied load and Berkovich tip [3].

The FSME images of the cross-sections of the UHTCs and the surfaces and cross-sections of the ablative UHTCs were, respectively, explored using FESEM (Nova NanoSEM 450, FEI Company, The Netherlands) assembled with an energy dispersive X-ray spectroscopy (EDXS). All samples were, respectively, sprayed with a thin layer of platinum before being examined using FESEM.

Grain size analysis of all UHTCs was conducted using “Image J Fiji 2.13.0” software.

The UHTCs were polished using an integrated automatic grinding and polishing machine with a programming control system (Tegramin-25, Struers, Ballerup, Denmark). Then, X-ray diffraction (XRD) data of the polished surface of the above UHTCs and the surface of the ablative UHTCs were, respectively, collected using XRD with the equipment and conditions described above.

Thermal gravimetric analysis (TGA) of the UHTCs were tested using a thermal analyzer (STA449F3, Neotchi Instrument Manufacturing Co. Ltd., Selb, Germany) with a heating rate of 20 °C/min up to 1450 °C in air.

Ablative testing of the polished UHTCs were conducted for 60 s using a welding torch (JH-3VA, Xiamen Jiarui Electronics Co., Ltd., Xiamen, China) equipped with a gas cylinder with gasses including methyl acetylene propylene propane (MAPP gas, Shanghai Chenmai Industrial Co., Ltd., Shanghai, China) that can shoot 1450 °C of flame in air. The samples were kept 7 cm away from the nozzle of the welding torch.

The linear ablation rate (R_l_) of the UHTCs was calculated according to the following formula [10]: R_l_ = (l_0_ − l_1_)/t, among which, l_0_ and l_1_ are the thickness of the samples at the center region before and after ablation, respectively; and t is an ablation time, which is 60 s.

The mass ablation rate (R_m_) of the UHTCs was calculated according to the following formula [15]: R_m_ = (m_0_ − m_1_)/(A·t), among which, m_0_ and m_1_ are the mass of the samples before and after ablation, respectively; A is the area of the front face of the samples; and t is an ablation time, which is 60 s.

## 3. Results and Discussion

### 3.1. Raw Material Microstructure

**Figure 1:**
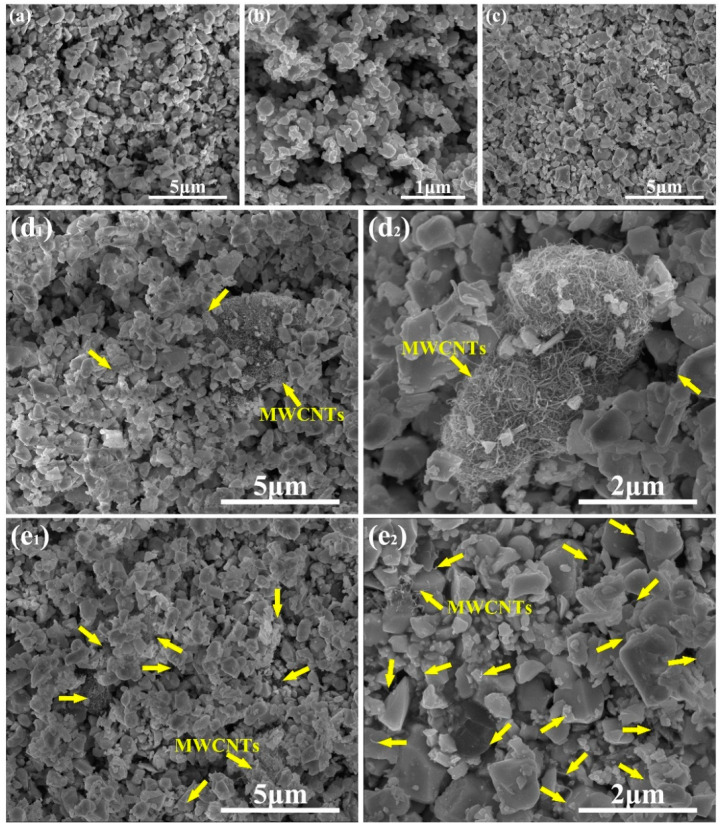
The microstructure of the pure TaB_2_ powder (**a**), pure TaC powder (**b**), 95TaB_2_-5TaC powder (**c**), 95TaB_2_-5TaC-0.5MWCNTs powder (**d_1_**,**d_2_**) and 95TaB_2_-5TaC-0.5MWCNTs-0.2PAA powder (**e_1_**,**e_2_**).

The microstructure of the pure TaB_2_ powder, pure TaC powder, 95TaB_2_-5TaC powder, 95TaB_2_-5TaC-0.5MWCNTs powder, and 95TaB_2_-5TaC-0.5MWCNTs-0.2PAA powder were, respectively, observed via FESEM, and the corresponding images are shown in Figure 1. From Figure 1a, the pure TaB_2_ powder is mostly irregular particles distributed in 1.0–3.0 μm range. The pure TaC powder is also mostly irregular particles with an average size of 300 nm (Figure 1b). From Figure 1(d_1_,d_2_), the aggregate MWCNTs with 5.0–10.0 μm are found in the 95TaB_2_-5TaC-0.5MWCNTs powder, while tiny aggregated and dispersed MWCNTs are observed in the 95TaB_2_-5TaC-0.5MWCNTs-0.2PAA powder (Figure 1(e_1_,e_2_)), disclosing that the use of PAA is beneficial to the dispersion of MWCNTs in the powders.

### 3.2. XRD Characterization for Raw Material

**Figure 2:**
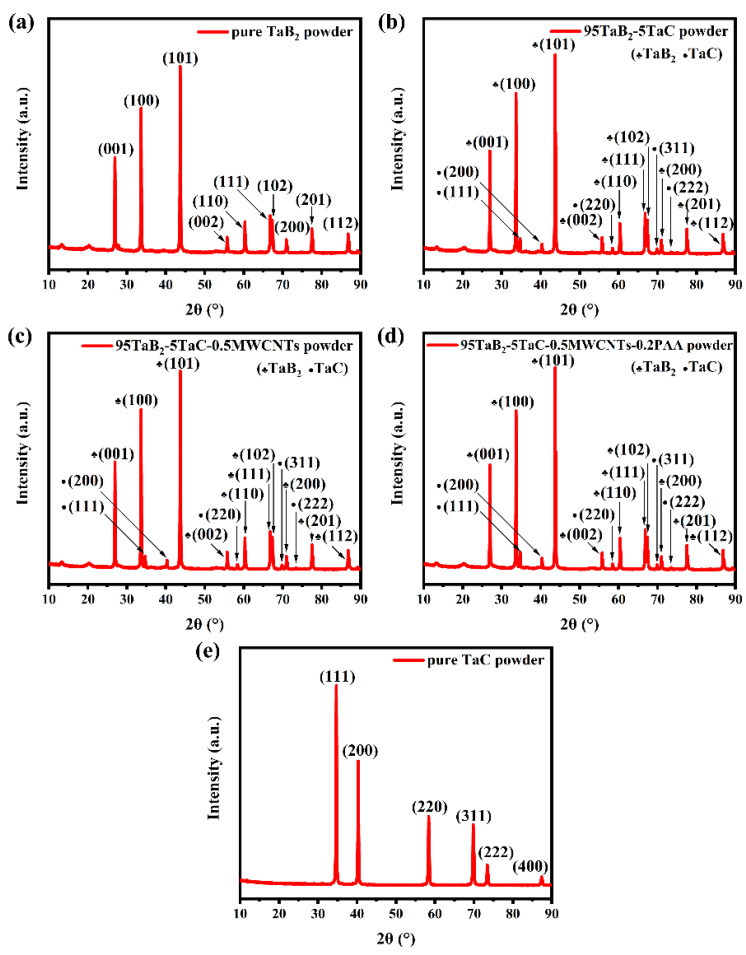
XRD curves of pure TaB_2_ powder (**a**), 95TaB_2_-5TaC powder (**b**), 95TaB_2_-5TaC-0.5MWCNTs powder (**c**), and 95TaB_2_-5TaC-0.5MWCNTs-0.2PAA powder (**d**), and pure TaC powder (**e**).

XRD data of the pure TaB_2_ powder, pure TaC powder, 95TaB_2_-5TaC powder, 95TaB_2_-5TaC-0.5MWCNTs powder, and 95TaB_2_-5TaC-0.5MWCNTs-0.2PAA powder were obtained using XRD, and the corresponding curves are present in Figure 2. From Figure 2a,e, the diffraction peaks at 27.5°, 33.5°,43.9°, 56.8°, 59.9°, 67.5°, 70.3°, 77.1°, and 87.1° are ascribed the crystal planes (001), (100), (101), (002), (110), (102), (200), (201), and (112) of TaB_2_ (PDF 75-0966) [16], consistent with the pure TaB2 powder, while the peaks at 35.0°, 40.6°, 58.7°, 70.2°, 73.8°, and 87.8° are related to (111), (200), (220), (311), (222), and (400) of the planes of TaC (PDF 65-8795) [17], in line with pure TaC powder. The strong peaks of TaB_2_ and the weak peaks of TaC were, respectively, detected in the 95TaB_2_-5TaC powder, 95TaB_2_-5TaC-0.5MWCNTs powder, and 95TaB_2_-5TaC-0.5MWCNTs-0.2PAA powder, revealing that TaC powder and TaB_2_ powder were mixed evenly during the powder processing, while neither peak of MWCNTs nor PAA was detected, possibly due to the low content of MWCNTs and PAA.

### 3.3. UHTCs’ XRD Characterization

**Figure 3:**
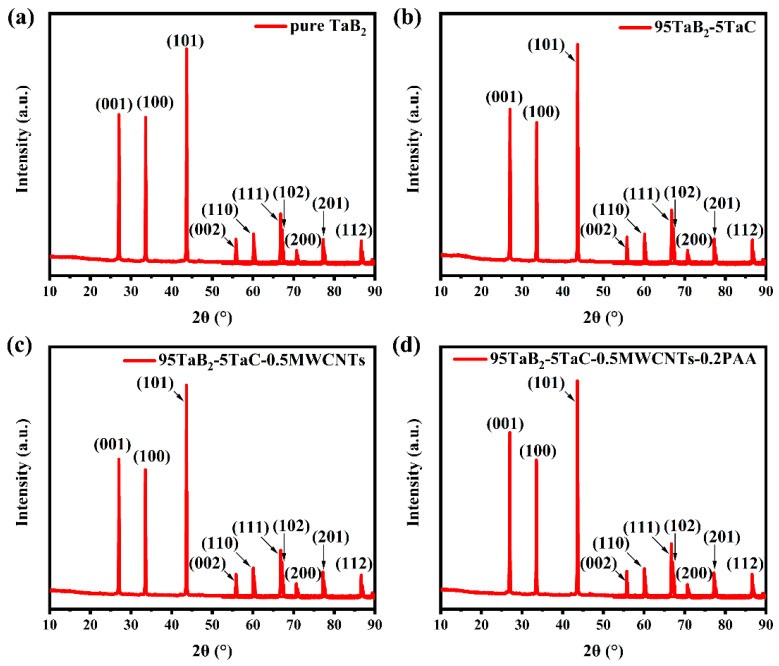
XRD curves of pure TaB_2_ (**a**), 95TaB_2_-5TaC (**b**), 95TaB_2_-5TaC-0.5MWCNTs (**c**), and 95TaB_2_-5TaC-0.5MWCNTs-0.2PAA (**d**).

XRD curves of the polished UHTCs are shown in Figure 3. Only the peaks of TaB_2_ were detected in all UHTCs’ XRD curves, while no peak of TaC was found. The reason for this could be that loose powders were more easily detected using XRD than dense ceramics at the low content (5.0 wt%) of TaC, which was consistent with the work of Akarsu [18].

### 3.4. UHTCs’ Cross-Sectional Microstructure

**Figure 4:**
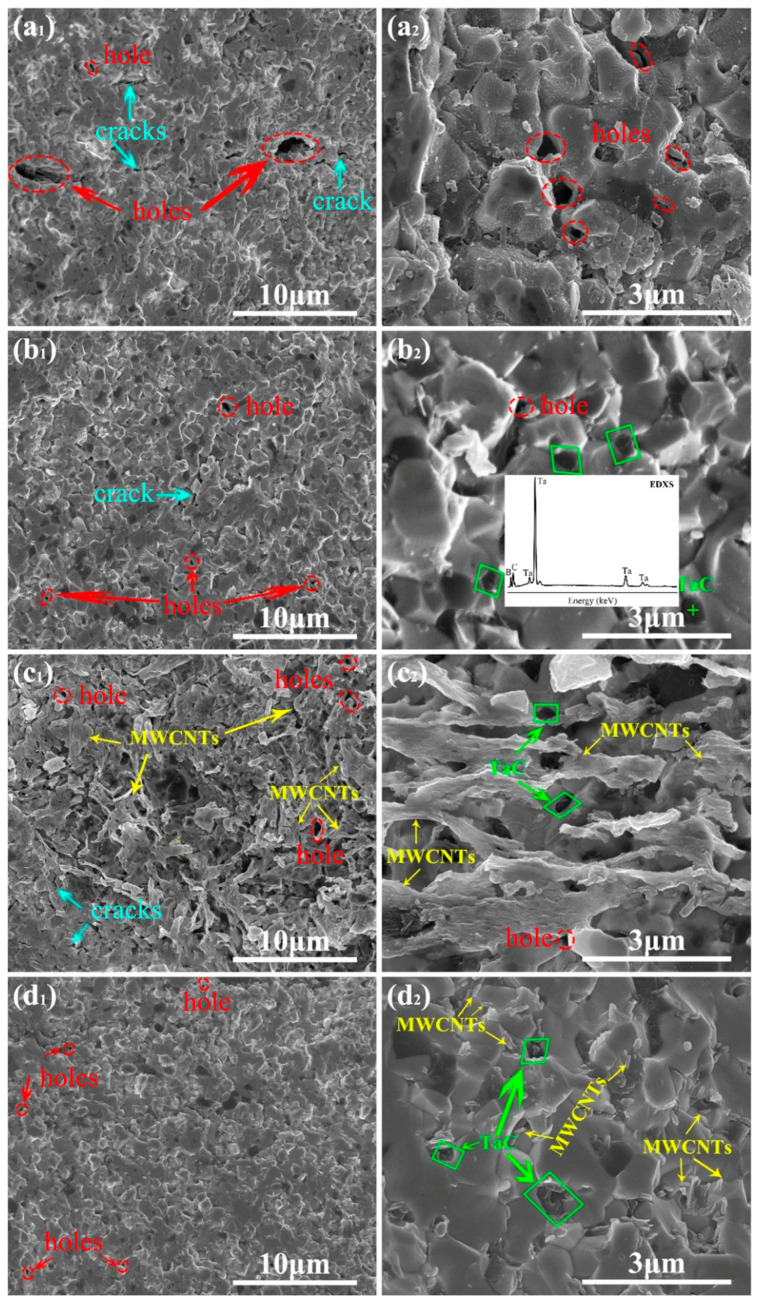
The microstructure of the pure TaB_2_ (**a_1_**,**a_2_**), 95TaB_2_-5TaC (**b_1_**,**b_2_**), 95TaB_2_-5TaC-0.5MWCNTs (**c_1_**,**c_2_**), and 95TaB_2_-5TaC-0.5MWCNTs-0.2PAA (**d_1_**,**d_2_**).

Before observing UHTCs’ cross-sectional microstructure using FESEM, the UHTCs were broken using a hammer. The corresponding FESEM images of their cross-sectional microstructure are presented in Figure 4. As shown in Figure 4(a_1_,a_2_), large holes and long cracks are found in the pure TaB_2_, owing to its relatively low sinterability. The average grain size of the pure TaB_2_ was 1.5 ± 0.2 μm, calculated using the “Image J Fiji” software. Compared to the microstructure of the cross-section of the pure TaB_2_, that of the 95TaB_2_-5TaC (Figure 4(b_1_,b_2_)) has fewer and smaller holes and cracks, and a finer average grain size (1.3 ± 0.2 μm), leading to an increased relative density for the 95TaB_2_-5TaC. The refinement of grain sizes was probably caused by the physical pinning effect of the small TaC particles. The physical pinning effect means that the distribution of the small TaC particles on the grain boundaries of TaB_2_ would slow down the rapid growth of the grains; similar phenomena also were reported in Refs. [16,19,20,21]. For example, Liu et al. [19] reported that the increasing amount of Si_3_N_4_ continuously reduced the grain size of TaC via physical pinning effect of Si_3_N_4_. Meanwhile, TaC was obviously discovered via EDXS, suggesting that TaC did not dissolve into TaB_2_ during the SPS processing of UHTCs. Previous researchers also reported similar results. Zhang et al. [22] found that TaC and TaB_2_ were virtually insoluble in each other below 2200 °C, which was assigned to the difference in the crystal structures. Particularly, TaC formed a cubic structure (B1); nevertheless, TaB_2_ crystallized in a hexagonal structure (AlB_2_ type) [18,22]. From Figure 4(c_1_,c_2_), the agglomerate MWCNTs are clearly observed, and the existence of holes and cracks in the ceramic matrix below MWCNTs are likely to be attributed to the three-dimensional structure of the agglomerate MWCNTs, which makes the powders difficult to compact via SPS. Therefore, the average grain size of the 95TaB_2_-5TaC-0.5MWCNTs is 1.4 ± 0.2 μm, a little larger than that of 1.3 ± 0.2 μm of the 95TaB_2_-5TaC. Fortunately, the holes of the 95TaB_2_-5TaC-0.5MWCNTs-0.2PAA are also very small, and the cracks could not be observed; meanwhile, the dispersed MWCNTs are seen on the boundaries of the grains or within the grains (seen Figure 4(d_1_,d_2_)). Owing to the physical pinning effect of smaller TaC particles and the sealing effect of the dispersed MWCNTs, the average gain size of the 95TaB_2_-5TaC-0.5MWCNTs-0.2PAA is further reduced to 1.3 ± 0.1 μm. The sealing effect of the dispersed MWCNTs referred to that MWCNTs wrapped around the grains of TaB_2_ and acted as barriers for mass diffusion, thereby inhibiting the grain growth during preparation of the TaB_2_-based UHTCs. The sealing effect was also reported in the previous studies. Li et al. [23] and Nieto et al. [24], respectively, reported that graphene platelet reduced the grains of the TaC-based UHTCs via the grain wrapping mechanism or the sealing effect. The microstructure refinement and the sealing effect led to the higher relative density, which was apt to enhance the mechanical, anti-oxidant, and anti-ablative properties for the UHTCs [19,25].

### 3.5. UHTCs’ Physical Properties

The data of UHTCs’ physical index, including average grain size, relative density, elastics modulus and nano hardness, are all summarized in Table 1. The average grain size of the pure TaB_2_, 95TaB_2_-5TaC, 95TaB_2_-5TaC-0.5MWCNTs, and 95TaB_2_-5TaC-0.5MWCNTs-0.2PAA, were respectively, 1.5 ± 0.2 μm, 1.3 ± 0.2 μm, 1.4 ± 0.2 μm, and 1.3 ± 0.1 μm. The decreased trend in the average grain size was ascribed to the physical pinning effect of smaller TaC particles and the sealing effect of the dispersed MWCNTs, as discussed above. The relative density (namely densification) of the pure TaB_2_ was 89.2% ± 0.8%, those of 95TaB_2_-5TaC, 95TaB_2_-5TaC-0.5MWCNTs, and 95TaB_2_-5TaC-0.5MWCNTs-0.2PAA were, respectively, increased to 95.9% ± 0.5%, 94.3% ± 0.7%, and 98.6% ± 0.4%, resulting from the gradual refinement of grain size and the excellent sealing effect. As known, the relative density and grain size both can crucially influence the mechanical properties of the UHTCs. Generally speaking, large relative density and refined grain size generate high elastic modulus and nano hardness for the UHTCs [3]. The pure TaB_2_ only had an elastic modulus of 356.3 ± 13.3 GPa and a nano hardness of 17.7 ± 1.4 GPa, while the 95TaB_2_-5TaC-0.5MWCNTs-0.2PAA possessed the highest elastic modulus (386.3 ± 9.6 GPa) and the biggest nano hardness (21.7 ± 1.0 GPa) among all UHTCs.

### 3.6. UHTCs’ TGA Characterization

**Figure 5:**
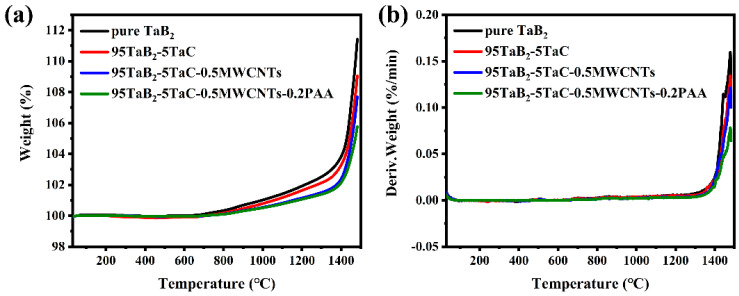
TGA curves (**a**) and DTG curves (**b**) of the UHTCs under air.

The oxidation behavior of the UHTCs was examined via TGA from room temperature up to 1450 °C in air with a heating rate of 20 °C/min. The possible reaction equations involved in the oxidation processes of the UHTCs are displayed in Equations (1)–(8). During the course of oxidation, TaC was oxidized to Ta_2_O_5_ and CO_2_; meanwhile, TaB_2_ was oxidized to Ta_2_O_5_ and B_2_O_3_. B_2_O_3_ melted at 450 °C and then vaporized above 1100 °C [10,26]. Although both CO_2_ and B_2_O_3_ were volatilized from the samples, the mass of the introduced oxygen element was greater than the added weight of both CO_2_ and B_2_O_3_, causing a gradual mass increase in the samples during the oxidation process. The TGA and DTG curves of the UHTCs are, respectively, displayed in Figure 5a,b. As shown in Figure 5a, the weight of the pure TaB_2_, 95TaB_2_-5TaC, 95TaB_2_-5TaC-0.5MWCNTs, and 95TaB_2_-5TaC-0.5MWCNTs-0.2PAA, respectively, accounts for 111.4%, 109.1%, 107.7%, and 105.8% after the oxidation procedure. From Figure 5b, the derivative weight of the pure TaB_2_, 95TaB_2_-5TaC, 95TaB_2_-5TaC-0.5MWCNTs, and 95TaB_2_-5TaC-0.5MWCNTs-0.2PAA is, respectively, 0.16%/min, 0.13%/min, 0.12%/min, and 0.08%/min. The results of TGA and DTG reveal that the 95TaB_2_-5TaC-0.5MWCNTs-0.2PAA has the best antioxidation property at higher temperature. The outstanding oxidation resistance for the 95TaB_2_-5TaC-0.5MWCNTs-0.2PAA is attributed to its high relative density and good sealing effect, as demonstrated in Figure 4(d_1_,d_2_).

4/9TaC (s) + O_2_ (g) = 2/9Ta_2_O_5_ (s) + 4/9CO_2_ (g) (1)

4/11TaB_2_ (s) + O_2_ (g) = 2/11Ta_2_O_5_ (s) + 4/11B_2_O_3_ (s) (2)

2x/(6x + 2y)TaB_2_ (s) + O_2_ (g) = 2/(6x + 2y)Ta_x_O_y_ (s) + 2x/(6x + 2y)B_2_O_3_ (s) (3)

B_2_O_3_ (s) → B_2_O_3_ (l) → B_2_O_3_ (g) (4)

1/7Ta_2_O_5_ (s) + C (s) = 2/7TaC (s) + 5/7CO (g) (5)

2TaC (s) + 2B_2_O_3_ (s) = 2TaB_2_ (s) + 2CO_2_ (g) + O_2_ (g) (6)

TaC (s) + B_2_O_3_ (s) = TaB_2_ (s) + CO (g) + O_2_ (g) (7)

2/11B_2_O_3_ (s) + 1/11Ta_2_O_5_ (s) + C(s) = 2/11TaB_2_ (s) + CO (g) (8)

### 3.7. Linear Ablation Rate and Mass Ablation Rate

Linear ablation rates (R_l_) and mass ablation rate (R_m_) are two vital indices for evaluating the ablation resistance of materials. The R_l_ and R_m_ of the polished UHTCs using a welding torch for 60 s are listed in Table 2. R_l_ of the pure TaB_2_, 95TaB_2_-5TaC, 95TaB_2_-5TaC-0.5MWCNTs, and 95TaB_2_-5TaC-0.5MWCNTs-0.2PAA were, respectively, −8.3 × 10^−2^ ± 2.7 × 10^−2^ μm/s, −5.0 × 10^−2^ ± 2.0 × 10^−2^ μm/s, −5.0 × 10^−2^ ± 2.1 × 10^−2^ μm/s, and −3.3 × 10^−2^ ± 1.1 × 10^−2^ μm/s. Their corresponding R_m_ were −2.0 × 10^−3^ ± 0.3 × 10^−3^ mg/(s·cm^2^), −1.7 × 10^−3^ ± 0.2 × 10^−3^ mg/(s·cm^2^), −1.8 × 10^−3^ ± 0.3 × 10^−3^ mg/(s·cm^2^), and −1.3 × 10^−3^ ± 0.2 × 10^−3^ mg/(s·cm^2^). The values of R_l_ and R_m_ for the ablative UHTCs were both negative, indicating that the mass of the UHTCs were increased during the ablation process due to the more introduced mass than the lost one, which was consistent with the results of TGA. The results of R_l_ and R_m_ revealed that the high relative density and good sealing effect were both beneficial to gaining the ablative resistance at high temperature for the 95TaB_2_-5TaC-0.5MWCNTs-0.2PAA.

### 3.8. XRD Characterization for Ablative UHTCs

**Figure 6:**
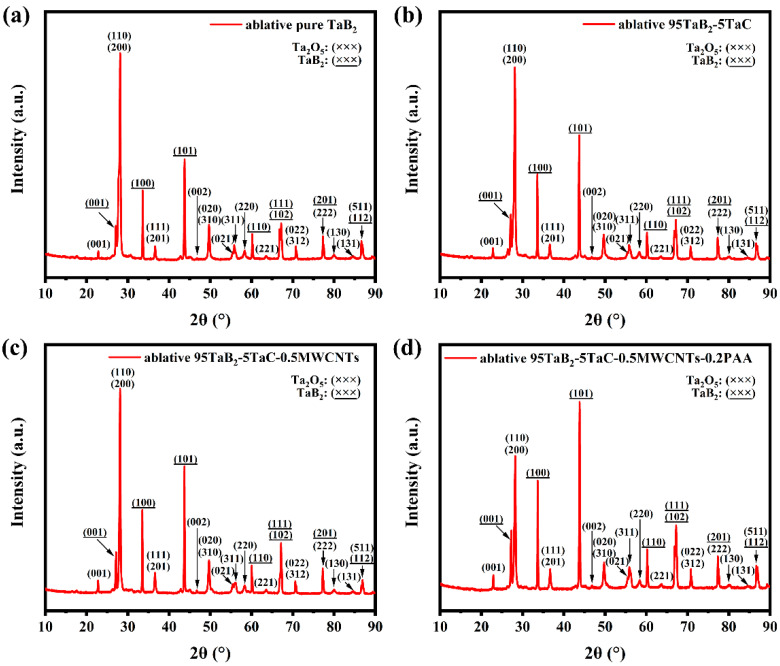
XRD curves of ablative pure TaB_2_ (**a**), ablative 95TaB_2_-5TaC (**b**), ablative 95TaB_2_-5TaC-0.5MWCNTs (**c**), and ablative 95TaB_2_-5TaC-0.5MWCNTs-0.2PAA (**d**).

The above ablative UHTCs were detected using XRD, and the corresponding XRD data are shown in Figure 6. From Figure 6, the peaks of both Ta_2_O_5_ and TaB_2_ are detected in all UHCTs. There were two possible reasons why the peak of TaB_2_ was detected. On the one hand, the shedding of the oxide layers resulted in the exposure of the un-oxidized ceramic matrix. On the other hand, the oxide layers were not thick enough (the thickness of oxide layers for all ablative UHTCs is shown in Figure 6), causing the ceramic substrate underneath the oxide layers to also not be detected. Notably, the detected oxide in the samples is Ta_2_O_5_ instead of other tantalum oxides because Ta_2_O_5_ has a higher melting point than other tantalum oxides [10]. As far as the peaks of B_2_O_3_ are not obviously detected, the reason is because the intensity of its peaks is much lower than that of Ta_2_O_5_, causing its peaks to be covered up, or there is not much B_2_O_3_ left, resulting in its peaks failing to be detected.

Compared to the pure TaB_2_, the maximum peak intensity ratio of Ta_2_O_5_ to TaB_2_ for 95TaB_2_-5TaC was decreased, indicating that 95TaB_2_-5TaC had a better ablation resistance due to its high relative density caused by the use of the finer TaC particles. Among all ablative UHCTs, the maximum peak intensity ratio of Ta_2_O_5_ and TaB_2_ for 95TaB_2_-5TaC-0.5MWCNTs-0.2PAA was the lowest, revealing that 95TaB_2_-5TaC-0.5MWCNTs-0.2PAA possessed the strongest ablation resistance because of its highest relative density and best sealing effect.

### 3.9. Ablative UHTCs’ Surface Microstructure

**Figure 7:**
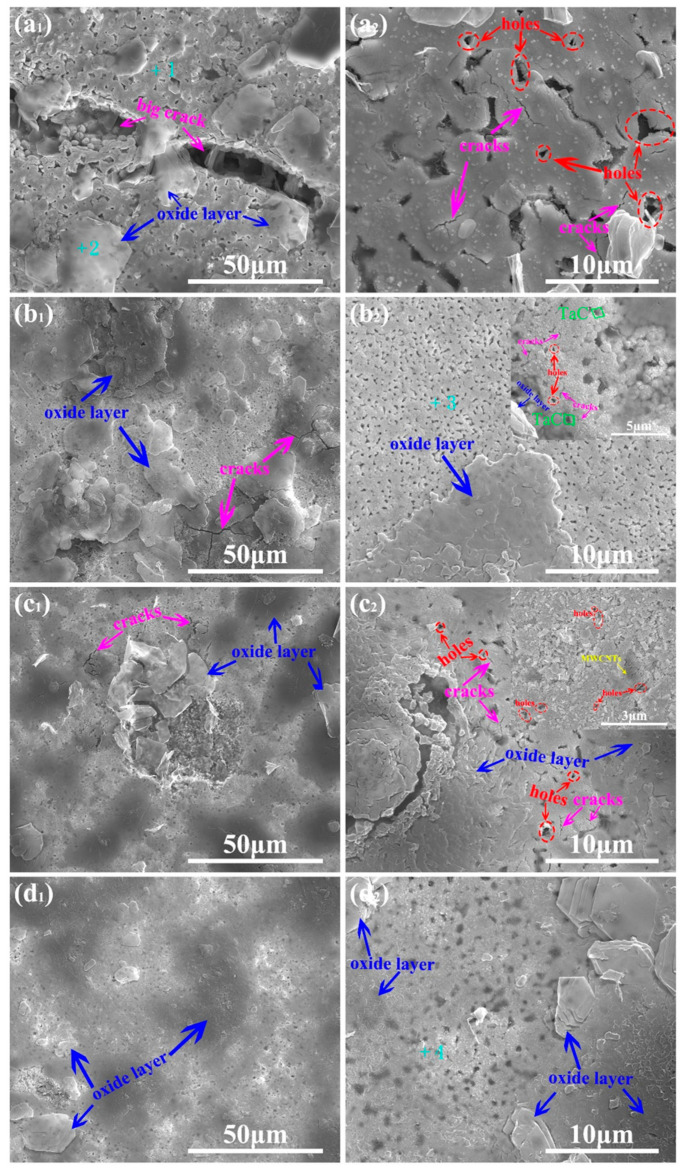
The surface microstructure of the ablative pure TaB_2_ (**a_1_**,**a_2_**), ablative 95TaB_2_-5TaC (**b_1_**,**b_2_**), ablative 95TaB_2_-5TaC-0.5MWCNTs (**c_1_**,**c_2_**), and ablative 95TaB_2_-5TaC-0.5MWCNTs-0.2PAA (**d_1_**,**d_2_**).

**Figure 8:**
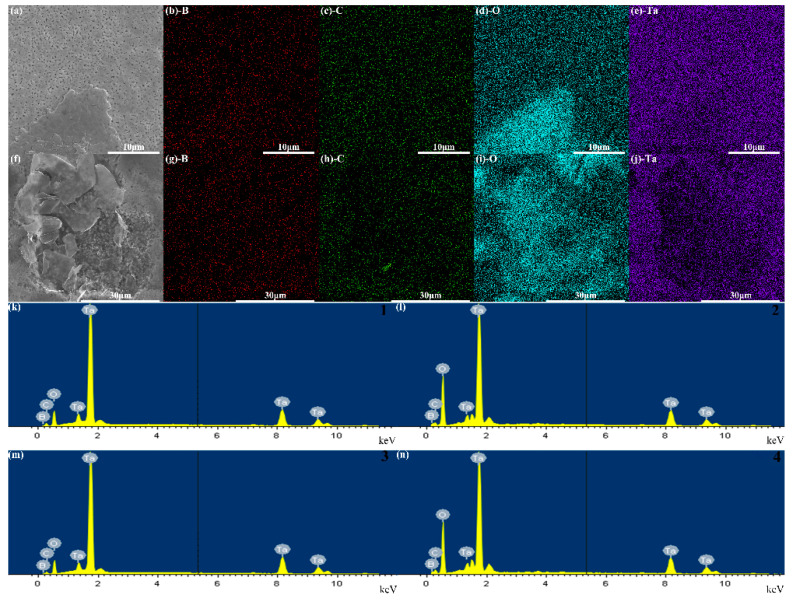
FESEM image (**a**) and EDXS elemental mapping (**b**–**e**) of the surface microstructure of the ablative 95TaB_2_-5TaC, FESEM image (**f**), and EDXS elemental mapping (**g**–**j**) of the surface microstructure of ablative 95TaB_2_-5TaC-0.5MWCNTs, with EDXS patterns of the selected regions in Figure 7 (**k**–**n**).

The ablative surface of the polished UHTCs was observed via an FESM equipped with an EDXS. The corresponding FESEM images and EDXS images of the ablative UHTCs’ surface microstructure are, respectively, shown in Figure 7 and Figure 8. As can be seen in Figure 7(a_1_,a_2_), a big crack and many small cracks, abundant scattered oxide layers, and a number of holes are observed in the surface of the ablative pure TaB_2_. The cracks and holes appeared for the following two reasons: one is because the cracks and holes are caused by the non-densification of the pure TaB_2_ matrix (see Figure 4(a_1_,a_2_)), and the other is because the volatilization of volatiles (such as CO, CO_2_, B_2_O_3_, etc., seen in Equations (1)–(8) above) resulted in the cracks and holes during the ablative process of the pure TaB_2_. The appearance of holes and cracks, especially cracks, were extremely unfavorable to the ablation resistance of ceramic matrix in the ablative process [27] because heat and oxygen could penetrate into the pure TaB_2_ matrix through holes and cracks, thus accelerating the oxidation of the pure TaB_2_. In comparison with Figure 7(a_1_,a_2_), no large cracks or holes are found in Figure 7(b_1_,b_2_), and the oxidation layers increased significantly and nearly became integral, but there still remained a few scattered oxide layers. In addition, there was an interesting phenomenon, being that TaC was found on the oxidized surface of the polished 95TaB_2_-5TaC. In general, the oxidation resistance of borides is better than that of carbides. Similarly, Zhang et al. [22] reported that the oxidation of TaC was faster than that of TaB_2_ using TGA in air up to 1500 °C. In order to further compare the high temperature oxidation resistance of TaC and TaB_2_ by calculating the free energy change per mole of oxygen (ΔG) and partial pressure of oxygen (*p*_O2_) of Equations (1) and (2), among which, ΔG reflects the feasibility of chemical reactions [28]. ΔG of the Equations (1) and (2) can be calculated by the following relationship (Equation (9)):

ΔG = −R·T·lnK_eq_ (9)

where R is the ideal gas constant, T is the absolute temperature, and K_eq_ is the equilibrium constant. The values of K_eq_ (with reaction numbers as subscripts) are presented as Equations (10) and (11), as follows:

(10) $$K_{1eq}=\frac{[(a_{Ta_{2}O_{5}}{)}^{2/9}\times \;(a_{{\mathrm{CO}}_{2}}{)}^{4/9}]}{[(a_{\mathrm{TaC}}{)}^{4/9}\times \;(p_{O_{2}})]}$$

(11) $$K_{2eq}=\frac{[(a_{Ta_{2}O_{5}}{)}^{2/11}\times \;(a_{B_{2}O_{3}}{)}^{4/11}]}{[(a_{{\mathrm{TaB}}_{2}}{)}^{4/11}\times \;(p_{O_{2}})]}$$

where *a* is the activity of the species and *p*_O_2__ is the equilibrium partial pressure. The values of ΔG and K_eq_ for 1450 °C (1723 K) calculated via Equation (9), as well as *p*_O_2__ calculated via considering the activities of pure substances as unity from Equations (10) and (11), are listed in Table 3. Table 3 shows the following: (i) ΔG_1_ < ΔG_2_, and (ii) the value of *p*_O_2__ required for the oxidation of TaB_2_ is tremendously less than that of TaC, displaying that the driving force of the oxidation at 1450 °C increases in the following order: TaC < TaB_2_. Via analyzing of thermodynamic data, TaC is more likely to be oxidized at higher temperature than TaB_2_. Hence, because of the probable reason that TaC existed in the oxide layers of the polished 95TaB_2_-5TaC, it is speculated that although TaC is easier to be oxidized thermodynamically, but due to the much lower content (5.0 wt%) of TaC and the extremely higher content (95.0 wt%) of TaB_2_, TaB_2_ is easily oxidized in exposure to air kinetically, resulting in that some TaC was wrapped by Ta_2_O_5_ products before being oxidized in the course of the crystallization growth of Ta_2_O_5_.

From Figure 7(c_1_,c_2_), in addition to cracks and holes, the agglomerated MWCNTs are also observed. As presented in Figure 7(d_1_,d_2_), the oxide layers are continuous, complete, and dense, and nearly no cracks and holes are found, disclosing that 95TaB_2_-5TaC-0.5MWCNTs-0.2PAA possesses a superior ablation resistance.

Figure 8 shows the EDXS images, including EDXS elemental mapping and EDXS patterns of the selected regions for the ablative UHTCs. According to Figure 8d, the retained oxide layers have higher oxygen content than that of the exposed substrates of 95TaB_2_-5TaC. Combined with Figure 8k–n, the oxygen element is found in both the oxide layers and the substrates, but its content in the substrates is relatively low, indicating that the ceramic substrates continue to be oxidized after the oxide layers fall off during the continuous ablation. 

### 3.10. Ablative UHTCs’ Vertical Cross-Sectional Microstructure

**Figure 9:**
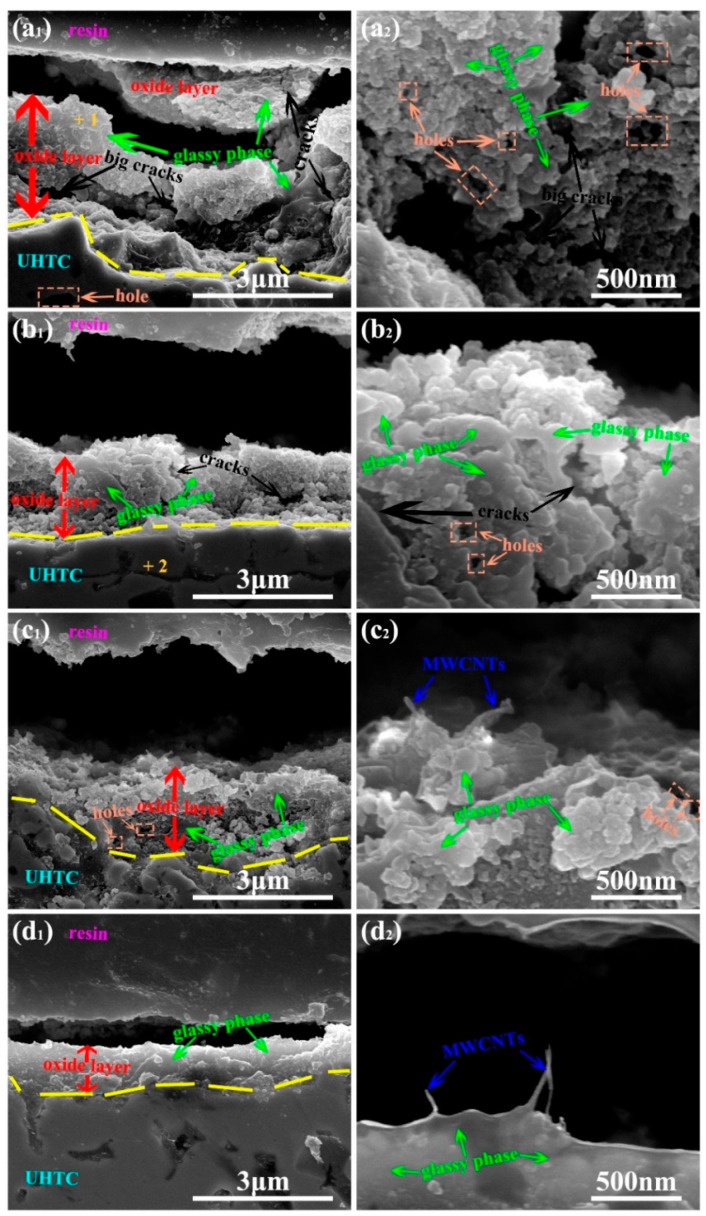
The vertical cross-sectional microstructure of ablative pure TaB_2_ (**a_1_**,**a_2_**), ablative 95TaB_2_-5TaC (**b_1_**,**b_2_**), ablative 95TaB_2_-5TaC-0.5MWCNTs (**c_1_**,**c_2_**), and ablative 95TaB_2_-5TaC-0.5MWCNTs-0.2PAA (**d_1_**,**d_2_**).

**Figure 10:**
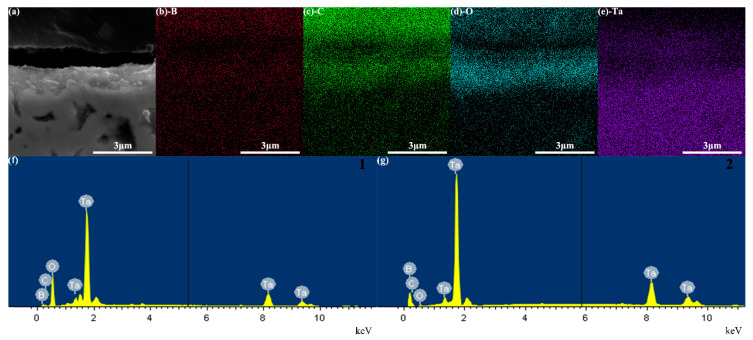
FESEM image (**a**) and EDXS elemental mapping (**b**–**e**) of the vertical cross-sectional microstructure of the ablative 95TaB_2_-5TaC-0.5MWCNTs-0.2PAA, with the EDXS patterns of the selected regions in Figure 9 (**f**,**g**).

After the ablation tests, the oxide layers of the ablative UHTCs’ vertical cross-sectional microstructure were also observed using FESEM, and the corresponding images are presented in Figure 9. From Figure 9(a_1_,a_2_), the oxide layer thickness of the ablative pure TaB_2_ is about 3.0 μm, while the penetration thickness of XRD equipment generally is up to tens of microns, which is the reason why the XRD curve of TaB_2_ presented both peaks of TaB_2_ and Ta_2_O_5_. The oxide layer of the ablative pure TaB_2_ appeared to have large cracks and even the separation between oxide layers, which consisted of many smaller grains of Ta_2_O_5_, were filled or covered by glassy B_2_O_3_. Compared to the surface of the ablative pure TaB_2_, the glassy B_2_O_3_ was detected in its vertical cross-section, because the temperature of UHTCs was dropped rapidly after the torch left and a certain amount of B_2_O_3_ remained in the oxide layers of the UHTCs before being volatilized [6]. It is noteworthy that a hole caused by the low sinterability of TaB_2_ was found in the pure TaB_2_ ceramic matrix, and a bright brim around the hole was detected due to higher second electron yield at the edge of the hole [19]. From Figure 9(b_1_,b_2_), the oxide layer about 2.0 μm thick, small cracks, as well as glassy B_2_O_3_, are found, finding that 95TaB_2_-5TaC possessed a better ablation resistance than that of pure TaB_2_. As shown in Figure 9(c_1_,c_2_), the oxide layer thickness of the ablative 95TaB_2_-5TaC-0.5MWCNTs is about 2.5 μm; meanwhile, a few MWCNTs, glassy B_2_O_3_, are also observed. The oxide layer thickness of the ablative 95TaB_2_-5TaC-0.5MWCNTs-0.2PAA is about 1.2 μm (see Figure 9d_1_), and the oxide layer is relatively dense, and MWCNTs are scarcely found in the oxide layer (see Figure 9d_2_). Figure 10 exhibits FESEM image and EDXS elemental mapping of the vertical cross-sectional microstructure of the ablative 95TaB_2_-5TaC-0.5MWCNTs-0.2PAA and the EDXS patterns of the selected regions in Figure 9, confirming that the oxygen content of oxide layers is higher than those of the non-oxide layers. By comparing the oxidation layers of all ablative UHTCs’ vertical cross-sections, it was found that 95TaB_2_-5TaC-0.5MWCNTs-0.2PAA had the best ablation resistance, which was consistent with the results of the ablative UHTCs’ surface microstructure. The excellent ablative resistance for 95TaB_2_-5TaC-0.5MWCNTs-0.2PAA was due to the dense oxide layer caused by the synergistic effect of the physical pinning of small TaC particles, the sealing of the dispersed MWCNTs, and the flowing B_2_O_3_ filled in the spaces of the grains of Ta_2_O_5_. The sealing of the dispersed MWCNTs mainly acted in three ways, namely, surface adsorption, melt wrapping, and reaction fusion (see Equations (5) and (8)) [29].

### 3.11. Ablation Mechanism of TaC and MWCNTs Reinforced TaB_2_-Based UHTCs

The ablation mechanism regarding transition metal-based UHTCs or transition metal-based ceramic composites was reported in the literature [7,14,25,30,31,32]. Generally speaking, the probable ablation mechanism can be summarized into three categories. The first is that small particles are utilized to fill the spaces between large raw material particles for preventing the excessive growth of ceramic grains through physical pinning for improving the relative density of ceramics in the ceramic preparation process, so as to play the role of ablative resistance. The second is that transition metal-based ceramics themselves or added secondary phases are oxidized into the flowing liquid oxides (such as B_2_O_3_, SiO_2_, and so on) for filling in the spaces of ceramic grains under the environment of high temperature and oxygen, in order to actively form the dense oxide layers retarding the transfer of heat and oxygen into the interior of the ceramic. The third is to use sealing materials, such as carbon nanotubes or graphene oxide, to seal the holes between ceramic grains and then promote the formation of relatively dense oxide layers for improving the anti-ablative performance of ceramics.

**Scheme 1:**
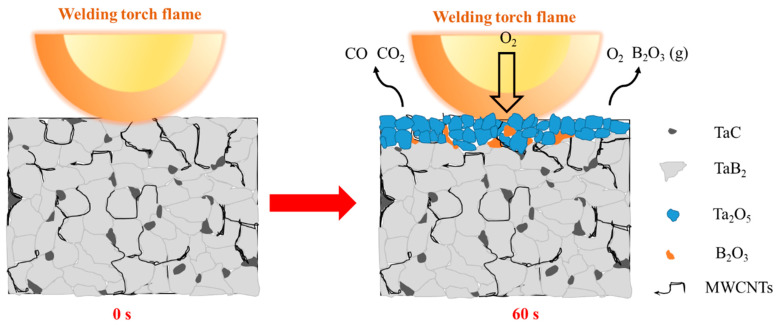
Schematic diagram of the ablative mechanism of the 95TaB_2_-5TaC-0.5MWCNTs-0.2PAA.

Scheme 1 presents the ablation mechanism of the 95TaB_2_-5TaC-0.5MWCNTs-0.2PAA, which includes all three ways to improve the ablation resistance. First, TaB_2_ was oxidized into forming liquid B_2_O_3_ and solid Ta_2_O_5_ at high temperature, and liquid B_2_O_3_ filled the spaces of solid Ta_2_O_5_ grains, generating the dense oxide layers. Second, fine TaC particles filled the spaces between TaB_2_ particles to promote the formation of the smaller ceramic grains and higher relative density, so as to enhance the ablation resistance. Third, MWCNTs were well dispersed in TaB_2_ and TaC powders by the dispersion of PAA to form 95TaB_2_-5TaC-0.5MWCNTs-0.2PAA with a good sealing for the ceramic holes and cracks, and then the good sealing impeded the diffusion of oxygen and heat into the interior of the ceramic matrix, thus resulting in further improving the ablation resistance. Hence, in addition to the flowing B_2_O_3_ filling in the spaces of the grains of Ta_2_O_5_, the fine TaC particles and the dispersed MWCNTs collaboratively further improved the ablation resistance of the TaB_2_-based HUTCs.

## 4. Conclusions

Four kinds of TaB_2_-based UHTCs were prepared via SPS at 1700 °C. Among them, 95TaB_2_-5TaC-0.5MWCNTs-0.2PAA possessed the optimal physical properties with a relatively small grain size of 1.3 μm, the maximal relative density of 98.6%, the largest elastics modulus of 386.3 GPa, and the highest nano hardness at 21.7 GPa, leading to the strongest oxidation resistance. Additionally, 95TaB_2_-5TaC-0.5MWCNTs-0.2PAA had the lowest linear ablation rate at −3.3 × 10^−2^ μm/s and the best mass ablation rate at −1.3 × 10^−3^ mg/(s·cm^2^), which is ascribed to the synergistic action of the self-healing effect originating from the flowing B_2_O_3_ filling the spaces between Ta_2_O_5_ grains, the physical pinning effect of fine TaC particles, and the sealing effect of the dispersed MWCNTs. The strategy for collaborative use of the fine TaC particles and the dispersed MWCNTs provides a helpful way for the preparation of ceramics with improved ablation resistance and physical properties.

## Figures and Tables

**Table 1:** A summary of average grain size, relative density, elastics modulus, and nano hardness of UHTCs.

Samples	Average Grain Size	Relative Density	Elastics Modulus	Nano Hardness
	μm	%	GPa	GPa
Pure TaB_2_	1.5 ± 0.12	89.2% ± 0.8%	356.3 ± 13.3	17.7 ± 1.4
95TaB_2_-5TaC	1.3 ± 0.2	95.9% ± 0.5%	372.1 ± 10.7	19.4 ± 1.2
95TaB_2_-5TaC-0.5MWCNTs	1.4 ± 0.2	94.3% ± 0.7%	368.1 ± 11.5	18.9 ± 1.4
95TaB_2_-5TaC-0.5MWCNTs-0.2PAA	1.3 ± 0.1	98.6% ± 0.4%	386.3 ± 9.6	21.7 ± 1.0

**Table 2:** Linear ablation rate (R_l_) and mass ablation rate (R_m_) of UHTCs.

Samples	R_l_ (μm/s)	R_m_ (mg/(s·cm^2^))
Pure TaB_2_	−8.3 × 10^−2^ ± 2.7 × 10^−2^	−2.0 × 10^−3^ ± 0.3 × 10^−3^
95TaB_2_-5TaC	−5.0 × 10^−2^ ± 2.0 × 10^−2^	−1.7 × 10^−3^ ± 0.2 × 10^−3^
95TaB_2_-5TaC-0.5MWCNTs	−5.0 × 10^−2^ ± 2.1 × 10^−2^	−1.8 × 10^−3^ ± 0.3 × 10^−3^
95TaB_2_-5TaC-0.5MWCNTs-0.2PAA	−3.3 × 10^−2^ ± 1.1 × 10^−2^	−1.3 × 10^−3^ ± 0.2 × 10^−3^

**Table 3:** Thermodynamic data of Equations (1) and (2) at 1450 °C (1723 K).

Reactions	△G (kJ/mol)	K_eq_	*p*_O_2__ (atm)
1	−400.7	6.4 × 10^11^	1.6 × 10^−12^
2	−476.2	1.1 × 10^14^	9.4 × 10^−15^

## Data Availability

The original contributions presented in the study are included in the article/Supplementary Material, further inquiries can be directed to the corresponding author.

## Supplementary Materials

The following supporting information can be downloaded at: https://www.mdpi.com/article/10.3390/ma17143394/s1, Figure S1: The SPS curves of pure TaB_2_, 95TaB_2_-5TaC, 95TaB_2_-5TaC-0.5MWCNTs and 95TaB_2_-5TaC-0.5MWCNT-0.2PAA; Figure S2: The top view (upper) and main view (lower) of pure TaB_2_ (A), 95TaB_2_-5TaC (B), 95TaB_2_-5TaC-0.5MWCNTs (C) and 95TaB_2_-5TaC-0.5MWCNTs-0.2PAA (D).
