# Supplementary material for: Enhancing Ablation Resistance of TaB2-Based Ultra-High Temperature Ceramics by Mixing Fine TaC Particles and Dispersed Multi-Walled Carbon Nanotubes
Source: Materials (Basel). 2024 Jul 9;17(14):3394. doi: 10.3390/ma17143394 (PMC11278642; doi:10.3390/ma17143394)
Supplement: Supplementary file 1 [file materials-17-03394-s001.zip › materials-3072854-supplementary.pdf]

Supplementary Information

Enhancing ablation resistance of TaB_2-based ultra-high temperature ceramics by mixing fine TaC particles and dispersed MWCNTs

Guangxu Bo[1], Xiaoke Tian[1], Huanhuan Li, Luona Ye, Xiaoling Xu, Zhaorui Gu, Jinyong Yan, Xingjian Su, Yunjun Yan*

Key Laboratory of Molecular Biophysics of the Ministry of Education, College of Life Science and Technology, Huazhong University of Science and Technology, Wuhan 430074, China；
B13991359@163.com (G.B.); D202280835@hust.edu.cn (X.T.); 18739976787@163.com (H.L.); yeln1992@163.com (L.Y.); xxl101010@126.com (X.X.); hustgzr@hust.edu.cn (Z.G.); yjiny@126.com (J.Y.); suxingjian@hust.edu.cn (X.S.)
*Correspondence: yanyunjun@hust.edu.cn
1 These authors contributed equally to this work.

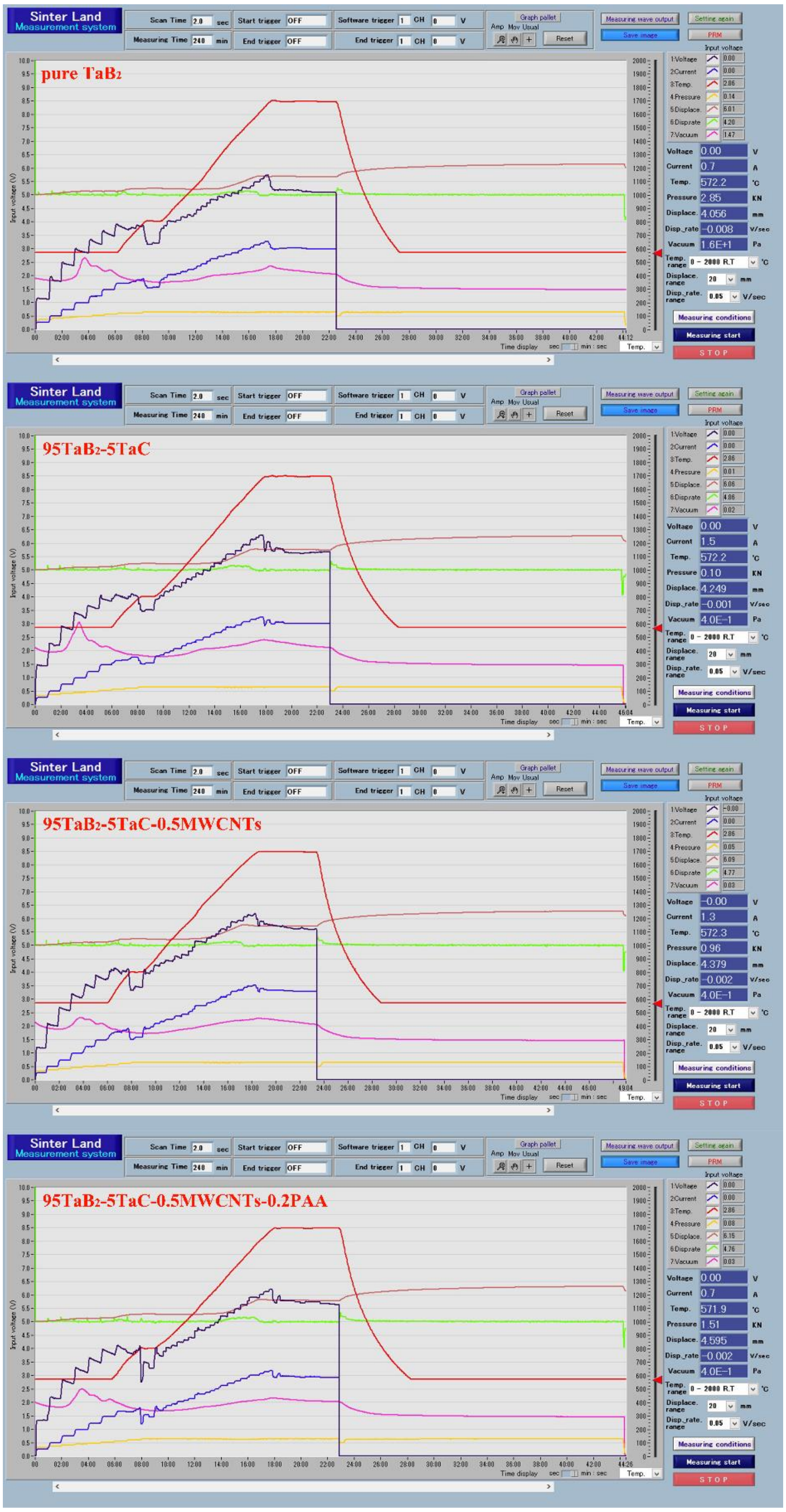

Figure S1. The SPS curves of pure TaB_2, 95TaB_2-5TaC, 95TaB_2-5TaC-0.5MWCNTs and 95TaB_2-5TaC-0.5MWCNT-0.2PAA.

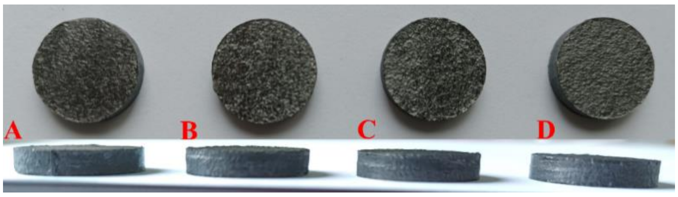

Figure S2. The top view (upper) and main view (lower) of pure TaB_2 (A), 95TaB_2-5TaC (B), 95TaB_2-5TaC-0.5MWCNTs (C) and 95TaB_2-5TaC-0.5MWCNTs-0.2PAA (D).
